# Economic Burden of Mosquito-Borne Diseases in Low- and Middle-Income Countries: Protocol for a Systematic Review

**DOI:** 10.2196/50985

**Published:** 2023-12-11

**Authors:** Nagavalli Chilakam, Varsha Lakshminarayanan, Sushanth Keremutt, Ambigai Rajendran, Girish Thunga, Pooja Gopal Poojari, Muhammed Rashid, Nirmalya Mukherjee, Paramita Bhattacharya, Denny John

**Affiliations:** 1 Department of Commerce Manipal Academy of Higher Education Manipal India; 2 Department of Pharmacy Practice Manipal College of Pharmaceutical Sciences Manipal Academy of Higher Education Manipal India; 3 Centre for Public Health Research Manbhum Ananda Ashram Nityananda Trust Kolkata India; 4 Faculty of Life and Allied Health Sciences MS Ramaiah University of Applied Sciences Bengaluru India; 5 School of Public Health The University of Adelaide Adelaide Australia

**Keywords:** cost of illness, economic burden, out-of-pocket expenditure, OOPE, mosquito-borne diseases, vector-borne diseases

## Abstract

**Background:**

Globally, among all the vector-borne diseases, mosquito-borne diseases are responsible for a substantial number of cases and deaths and amount to an economic cost of US $12 billion per year. However, there is a dearth of systematic research conducted on the economic burden of mosquito-borne diseases. To address the lack of comprehensive information on this topic, a systematic review will be conducted to synthesize evidence for informing targeted policies and strategies addressing this growing burden and for better financial protection of households.

**Objective:**

The systematic review aims to review the economic burden of mosquito-borne diseases in low- and middle-income countries (LMICs). The review estimates the total cost, which is the compilation of both the direct costs and indirect costs. Additionally, it reports cost estimates per disease, country, and patient. The review outcome will also discuss the impact of the economic burden in terms of out-of-pocket expenditure, catastrophic health expenditure, impoverishment, and gross domestic product impact due to mosquito-borne diseases in LMICs.

**Methods:**

Systematic searches will be conducted in PubMed (MEDLINE), Ovid Embase, Scopus, the cumulative index of nursing and allied health literacy, and Cochrane CENTRAL. Additionally, websites of the World Bank, World Health Organization, and Asian Development Bank as well as grey literature (eg, Malaria No More and the Ministry of Health websites) will be searched to gather comprehensive information on the topic and identify studies published in the English language. The titles and abstracts will be independently screened by 2 reviewers, followed by a full-text review against the inclusion criteria. Disagreements will be resolved through discussion with a third author. The methodological reporting quality of the studies will be evaluated using the Larg and Moss checklist, Cochrane risk-of-bias tool for randomized trials, and the Consensus on Health Economic Criteria. Data will be extracted using a standardized data extraction form.

**Results:**

The protocol was registered in PROSPERO (CRD42023427111) prior to the initiation of the search strategy. The review is currently ongoing and will synthesize information from the identified studies through a process involving structured screening, data extraction, and critical appraisal in the form of tables and a narrative summary of studies reporting the economic burden incurred due to mosquito-borne diseases in LMICs.

**Conclusions:**

This review seeks to report the economic burden of mosquito-borne diseases. It will act as evidence for policymakers to prioritize their decisions regarding containing the prevalence of mosquito-borne diseases and the means to lowering the incidence of diseases spread by mosquitoes.

**Trial Registration:**

PROSPERO CRD42023427111; https://www.crd.york.ac.uk/prospero/display_record.php?RecordID=427111

**International Registered Report Identifier (IRRID):**

PRR1-10.2196/50985

## Introduction

### Background

Vector-borne diseases are diseases transmitted to humans by vectors (eg, aquatic snails, mosquitos, fleas, blackflies, ticks, lice, sandflies, triatome bugs, and tsetse flies) infected either with viruses, parasites, or bacteria. These account for greater than 17% of all infectious diseases and play a huge role in the economic and disease burden. Among these vectors, mosquito-borne diseases are responsible for almost 700 million cases and more than a million deaths each year. The most important mosquito vectors are *Aedes*, *Anopheles*, and *Culex*, which are responsible for the diseases chikungunya, lymphatic filariasis, dengue, Rift Valley fever, Zika, malaria, yellow fever, West Nile fever, and Japanese encephalitis [1,2].

Every year, more than 1 million people die after contracting a mosquito-borne disease. The US Centers for Disease Control and Prevention reported that more than 1 million people die because of malaria in a year. The pace of infection dissemination has substantially increased during the past few years [3]. It has been estimated that 3.4 billion people are in danger of contracting malaria, which is spreading in 97 countries [4]. The frequency of dengue has increased 30-fold over the past 50 years, and the disease has begun to move from urban to rural areas [5]. As stated by the World Health Organization, there are 50 to 100 million cases of dengue, an arboviral disease, per year [6]. *Aedes albopictus* and *Aedes aegypti* are the primary vectors of dengue [7]. Yellow fever has a high fatality rate, and many patients need supportive care to manage the fever, respiratory failure, and dehydration [8]. Around 10,000 people every year die from Japanese encephalitis, predominantly in Asia [9].

The estimated total economic burden of lymphatic filariasis was US $5.8 billion annually [10]. For the period 2011-2020, the total productivity loss was US $10.5 billion, and for 2021-2030, the anticipated loss is US $13.8 billion [11]. Dengue fever was reported to have a US $8.9 billion yearly global cost in 2013 [12]. The estimated socioeconomic impacts of Rift Valley fever ranged from US $5 million to US $470 million [13]. It has been estimated that the Zika virus costs the global economy a total of US $8.9 billion [14]. In 2016, the total cost of malaria worldwide, including both government and out-of-pocket expenditure (OOPE), was US $4.3 billion [15]. Mosquito-borne diseases contribute to acute and long-term complications, thus affecting the gross domestic product (GDP) of a country.

Nations suffer the loss of billions of dollars annually by investing in treatments for mosquito-borne diseases. For example, households lose an estimated 25% of their income to OOPE for treating malaria, treatment-related travel, and other diagnosis-related expenses, which amount to an average cost of US $3 per case [16]. Most low- and middle-income countries (LMICs) have many cities with high-density populations in urban areas that are associated with poor hygiene and poor water quality, which are characteristics that lead to increased mosquito breeding and the rapid spread of mosquito-borne diseases. Most LMICs have limited universal health coverage (UHC) due to low public health investments, resulting in a substantial proportion of the population being exposed to OOPE, with some of these populations experiencing catastrophic health expenditure and impoverishment.

A preliminary systematic search of the PROSPERO (International Prospective Register of Systematic Reviews), PubMed (MEDLINE), Cochrane Database of Systematic Reviews, Joanna Briggs Institute (JBI) Evidence Synthesis, and Epistemonikos databases identified no published or ongoing systematic reviews on the topic. Systematic reviews on the economic burden of individual diseases (eg, dengue and malaria) have been conducted previously [15,17-19]. However, these reviews have either focused on a specific country or a specific disease and did not consider other mosquito-borne diseases. To fill this information gap, a systematic review of the literature on the economic burden on households and countries due to mosquito-borne diseases in LMICs will be performed. The primary objective is to synthesize available evidence on costs (direct medical, direct nonmedical, and indirect), resources used, and health care expenditure due to mosquito-borne diseases in LMICs. Financial protection is at the core of UHC, as outlined in Sustainable Development Goal 3.8.2 related to indicators of financial hardship, such as catastrophic health expenditure and poverty impact [20]. The review will help to evaluate currently available evidence on OOPE, catastrophic health expenditure, impoverishment, and GDP impact due to mosquito-borne diseases in LMICs. Hence, our review findings will be crucial toward framing targeted policies focusing on increased prevention and health protection strategies to address the growing burden of mosquito-borne diseases in LMICs.

### Operational Definitions

The following are operational definitions pertinent to the systematic review:

- Chikungunya: “Chikungunya fever is an acute febrile illness caused by an arthropod-borne alphavirus, Chikungunya virus (CHIKV). The virus is primarily transmitted to humans via the bite of an infected *Aedes* species mosquito” [21].
- Dengue: “Dengue (break-bone fever) is a viral infection that spreads from mosquitoes to people. It is more common in tropical and subtropical climates” [6].
- Lymphatic filariasis: “Lymphatic filariasis, often known as elephantiasis, is a human infection that is caused by the transmission of parasites called filarias through mosquitoes, including those of the genus *Culex*, which is widespread in urban and semi-urban areas” [22].
- Rift Valley fever: “Rift Valley fever is a neglected, mosquito-borne and direct contact viral zoonosis associated with significant morbidity, mortality and an expanding geographical scope” [23].
- Yellow Fever: “Yellow fever (YF) is a viral acute hemorrhagic illness caused by infected mosquitoes of the flavivirus family” [24].
- Zika: “Zika is spread mostly by the bite of an infected *Aedes* species mosquito (*Ae. aegypti* and *Ae. albopictus*). These mosquitoes bite during the day and night” [25].
- Malaria: “Malaria is a parasitic infection transmitted by the *Anopheles* mosquito that leads to acute life-threatening disease and poses a significant global health threat” [26].
- Japanese encephalitis: “Japanese encephalitis (JE) is a vector-borne disease caused by the Japanese encephalitis virus (JEV), which is a single-stranded ribonucleic acid (RNA) virus belonging to the genus *Flavivirus* (Flaviviridae family) and is closely related to the West Nile encephalitis virus” [27].
- West Nile fever: “West Nile fever is an acute febrile illness associated with malaise, rash, headache, myalgia, and lymphadenopathy. Infection involves a bird-mosquito-human cycle” [28].
- Economic burden: “Economic burden captures the economic impact of a disease or illness of interest on both the health sector and non-health sectors at both microeconomic and macroeconomic levels. Economic burden is defined by cost-of-illness studies that estimate direct and indirect costs due to disease” [29].
- Direct costs: “Direct costs are defined as costs related to the use of resources due to either the disease or its treatment. Direct costs include costs to the health care system (direct medical costs) and costs to social services and to patients themselves or to their relatives (direct non-medical costs)” [30].
- Indirect costs: “Indirect costs or loss of production are defined as costs that occur to society related to loss of production, due either to the disease or its treatment” [30].
- Catastrophic health expenditure: “Population with household expenditures on health greater than 10% of total household expenditure or income” [31].
- Financial hardship: “Households incur ‘hardship financing’ when they are exposed to a less stable or worsened financial state brought about by additional costs/losses due to borrowing or selling assets” [32]. “Out-of-pocket (OOP) health care payments financed through borrowings or sale of household assets are referred to as distressed health care financing” [33].
- Impoverishment: “Impoverishment occurs when a ‘non-poor’ household becomes ‘poor’ after paying for health services” [34].

### Review Question

The systematic review seeks to determine the economic burden associated with mosquito-borne diseases in LMICs by answering the following questions: (1) What are the direct and indirect costs associated in treating mosquito-borne diseases in LMICs? (2) What is the impact of the economic burden in terms of OOPE, catastrophic health expenditure, impoverishment, and GDP due to mosquito-borne diseases in LMICs? (3) What is the per patient cost involved in the treatment of mosquito-borne disease? The review will also aim to present evidence across specific mosquito-borne diseases and across all specific LMICs.

## Methods

### Reporting

The PRISMA-P (Preferred Reporting Items for Systematic Reviews and Meta-Analyses Protocols) was used to develop the protocol and will follow the JBI protocols for economic evaluations [35,36]. The final systematic review will be undertaken using the JBI methodology for economic evaluations and reported as per PRISMA 2020 guidelines [37,38].

### Inclusion Criteria

#### Participants

This review will consider studies that include patients of all age groups who are affected with mosquito-borne diseases and residing in LMICs.

#### Conditions

This review will focus on studies that report the costs of the population affected with chikungunya, lymphatic filariasis, dengue, Rift Valley fever, Zika, malaria, yellow fever, West Nile fever, and Japanese encephalitis.

#### Contexts

This review will include studies conducted in any setting and in any geographical location in LMICs as per the World Bank criteria (Multimedia Appendix 1). For the current 2024 fiscal year, low-income economies are defined as those with a gross national income per capita of US $1135 or less in 2022, and lower middle-income economies are those with a gross national income per capita between US $1136 and US $4465, as calculated using the World Bank Atlas method. Annexure provides the list of LMICs included in the review [39]. 

#### Study Designs

This systematic review will consider study designs published in English that involve full or partial economic evaluation, which may include cost analysis, cost comparison, cost of illness or economic burden, cost effectiveness, cost utility, cost benefit, and cost minimization studies. All studies that focus on the economic burden of vector-borne diseases spread by mosquitos in LMICs will be included as per the World Health Organization’s definition.  

### Exclusion Criteria

The following studies were excluded from review: (1) studies with costs pertaining to the comorbidities associated with mosquito-borne illnesses; (2) studies that only report utilities, quality of life, and patient-related outcomes without costs; (3) editorials, methodological articles or commentaries, and reviews (however, the bibliographies of systematic reviews will be used to identify relevant studies to be included); (4) studies conducted in high-income countries; and (5) studies published in a language other than English.

### Outcomes

The outcomes of this review will consist of metrics relating to cost components that contribute to the economic burden of mosquito-borne diseases.   

#### Measures of Costs

Cost outcomes will be expressed as total costs per patient, per country, and per disease as per the data available in the included studies. Costs will be categorized as direct or indirect as per the availability of the data.

#### Measures of Resource Use

The costs associated with clinical outcomes (recovery, morbidity, and mortality) will be measured in terms of the total number of units of resources used. Resources used will be measured in 3 categories. Direct medical resources include consultations, number and length of outpatient visits, hospitalization days, provider services, devices, drugs, supplies, procedures (laboratory and clinical), and investigations. Direct nonmedical resources include the number or duration of services (housekeeping and administration), transportation, accommodation, food, clothing, overhead costs, waiting time, and property losses. Indirect resources include the number of days or hours of work lost due to the disease and productivity loss by patients and their caregivers due to morbidity, disability, or premature death.

#### Measures of Health Care Expenditure

Health care expenditure includes costs reported in terms of OOPE and the percentage of the population exposed to catastrophic health expenditure, financial hardship, impoverishment, and GDP impact.

### Search Strategy

The first step is to develop a search strategy that will include combinations of medical subject heading (MeSH) terms and free texts. Search terms will be developed for the domains, mosquito-borne diseases (conditions), costs (outcomes), and LMICs (contexts). The search terms for mosquito-borne diseases will include the following MeSH terms: “chikungunya,” “lymphatic filariasis,” “dengue,” “Rift Valley fever,” “Zika,” “malaria,” “yellow fever,” “West Nile fever,” and “Japanese encephalitis.” The MeSH terms for the costs will include “economics,” “cost,” and “cost analysis.” The MeSH term for the LMICs will be “developing countries.” The search filters for costs and LMICs were adapted from Canada’s Drug and Health Technology Agency search filters database [40] and the School of Health and Related Research LMIC filter [41], respectively.

These search terms will be combined using the “OR” and “AND” Boolean operators. “OR” will be used to combine search terms within a particular domain, and “AND” will be used to combine different domains. In the next step, the search strategy used in PubMed (MEDLINE) will be peer reviewed following the Peer Review of Electronic Search Strategies 2015 guideline statement [42]. After the search strategy is peer-reviewed, corrections will be made for any suggestions or errors. Once the search strategy is updated, the same will be replicated in all other databases included. No specific date range will be applied (Multimedia Appendix 2).

The search strategy will include databases such as PubMed (MEDLINE), Ovid Embase, Scopus, the cumulative index of nursing and allied health literacy, and Cochrane CENTRAL. Additionally, the websites of the World Bank, World Health Organization, and Asian Development Bank will also be searched. Grey literature will also be identified through a manual search of relevant organizations working in the field, such as Malaria No More, and Ministry of Health websites.

### Study Selection

All references retrieved through searches will be imported into Zotero and duplicates will be removed. Following a pilot test to check for interrater reliability, titles and abstracts will be screened by 2 independent reviewers for assessment against the inclusion criteria for the review. Potentially relevant studies will be retrieved in full, and their citation details will be imported into Covidence software [43]. The full text of selected citations will be assessed in detail as per the inclusion criteria by 2 independent reviewers (VL and NC). Reasons for the exclusion of full-text studies that do not meet the inclusion criteria will be recorded and reported in the systematic review. Any disagreements that arise between the reviewers at each stage of the study selection process will be resolved through discussion or adjudication by a third reviewer (SK).

The results of the search and study selection and inclusion process will be reported in full in the final systematic review and presented in a PRISMA flow diagram [38].

### Assessment of Methodological Quality

The methodological quality of the included studies will be individually evaluated by 2 authors (VL and NC) and then reviewed independently by 2 other authors (SK and AR). When necessary, the authors of the included papers will be contacted for clarification on any missing information or additional data. The tools used will include an adapted checklist by Larg and Moss [44] for cost of illness studies, the Cochrane risk-of-bias tool (RoB 2) [45] for randomized controlled trials, and the Consensus on Health Economic Criteria [46] for economic evaluation studies. Data extraction and synthesis will be applied to all studies, regardless of their methodological quality.

### Data Extraction

Data extraction will be performed in Excel (Microsoft Corp) using a standardized form (Multimedia Appendix 3). The form will be pilot tested by 3 independent authors (VL, NC, and SK) and reviewed by 2 additional authors (AR and DJ). It will then be used to collate relevant information from the included studies and will be tested by all authors. Data extraction will be performed by 3 independent authors (VL, NC, and SK), and any disagreements will be resolved through discussion with a senior reviewer (AR or GT). Where required, authors will be contacted for any missing or additional information.

### Data Synthesis

The studies selected for review will be presented in tables and a narrative summary. The summary will evaluate and compare the methods and primary outcomes across the studies and will be organized according to the country where the study was published, timing, intervention type, economic evaluation type, and the methodology used. The studies will be categorized according to the kind of economic assessment conducted, sources of data, effect size of the intervention, resources usage, costs, epidemiological data, and effectiveness estimates. Further, to facilitate comparison of the studies, they will be summarized in a table. The table will facilitate the collection of information on the year of analysis, currency, type of intervention, and costs reported by the authors of the studies. A table named “characteristics of the included studies” will be created to present the methodological features of the studies included in the review. 

For comparison across studies, all costs will be converted into a single currency (USD) and reference year (2022) using the cost calculator provided by the Campbell and Cochrane Economics Methods Group and the Evidence for Policy and Practice Information Centre [47].

## Results

The protocol has been registered in PROSPERO. The systematic review is self-funded and was started on May 20, 2023. A systematic search was conducted without any restriction on time period by adapting search filters for economic evaluations from Canada’s Drug and Health Technology Agency search filters database and the School of Health and Related Research LMIC filter in May 2023. The title and abstract screening of 13,444 studies was conducted, and 267 studies met the criteria for full text review. Data extraction is currently underway. After data synthesis, we intend to disseminate the results through the submission of a manuscript to a peer-reviewed journal by November 2023. The systematic review conducted as per this protocol will present the results evaluating and comparing the methods and primary outcomes across the included studies and will be presented in the form of tables and a summary.

## Discussion

### Overview

Mosquito-borne diseases pose a significant economic burden in LMICs. Irrespective of age, race, and income level, anyone can become affected by these diseases. The systematic review will comprehensively synthesize all the existing literature on the economic burden of mosquito-borne diseases and will provide an estimate of their total economic burden. The gap in the existing literature [15,17-19]—that studies have concentrated only on either a specific disease or a specific country—will be addressed in this review. The review’s findings will compile the direct and indirect costs associated with treating mosquito-borne diseases. Also, it will offer estimates on the total economic burden caused by mosquito-borne diseases in specific LMICs. Furthermore, the review will determine the disease contributions in the accumulation of economic burden specific to LMICs, as well as per patient cost estimates involved in the treatment of mosquito-borne diseases.

Due to the low number of populations with health care coverage, OOPE has increased considerably in LMICs. This has significantly increased the economic burden on individuals living in LMICs. The outcome of the review will help governments and international bodies to prioritize decisions about how to contain the prevalence of mosquito-borne diseases and inform actions to lower the incidence of diseases spread by mosquitoes.

The most significant limitation of this systematic review will be the necessity that each study included in the review be a full-text, primary study, as conference abstracts will be excluded. Another limitation will be that only studies in English will be included. However, we do not foresee any issues due to these limitations since conference abstracts provide limited information with regard to the methods of economic burden estimation and do not provide sufficient information for conducting a proper critical appraisal using standard tools. Also, the review will not include studies regarding the prevention of mosquito-borne diseases. Studies published in regional language databases, for example Spanish and Chinese language databases, will be excluded; however, this will not have a large impact on the number of studies that will be excluded due to this criterion.

### Conclusion

The purpose of this review is to explore the extent of the literature available on mosquito-borne diseases in LMICs. As reported in the literature, given that the burden of mosquito-borne diseases in LMICs is high, a comprehensive review will help to explore the economic burden associated with these diseases in this context. The review outcome will reveal the economic burden caused by mosquito-borne diseases in specific countries and LMICs on the whole. Further, based on the available studies, the review will also identify specific diseases that are increasing the economic burden of individual LMICs. The review will help to evaluate the currently available evidence on OOPE, catastrophic health expenditure, impoverishment, and GDP impact due to mosquito-borne diseases in LMICs. Hence, our review findings will help frame targeted policies focusing on decisions for containing the prevalence of mosquito-borne diseases and inform a means to lower the incidence of diseases spread by mosquitoes. The review will also guide future researchers in designing country-specific studies by choosing an appropriate mosquito-borne disease that increases the economic burden. Also, based on the findings of our full economic evaluation study, future researchers can engage in treatment-based cost-effectiveness studies based on country-specific treatment protocols.

## Appendix

Multimedia Appendix 1 World Bank list of low- and middle-income countries.

Multimedia Appendix 2 Search strategy.

Multimedia Appendix 3 Data extraction form.

## Abbreviations

CCEMG: Campbell and Cochrane Economics Methods Group
CHIKV: chikungunya virus
GDP: gross domestic product
JBI: Joanna Briggs Institute
LMIC: low- and middle-income countries
MeSH: medical subject headings
OOPE: out-of-pocket expenditure
PRISMA: Preferred Reporting Items for Systematic Reviews and Meta-Analyses
PROSPERO: International Prospective Register of Systematic Reviews
UHC: universal health coverage

## Data Availability

Our review is ongoing; therefore, we do not currently have a data set to share. As our study period ends in November 2023, we can share the data with the readers on request thereafter.
