# Supplementary material for: Economic Burden of Mosquito-Borne Diseases in Low- and Middle-Income Countries: Protocol for a Systematic Review
Source: JMIR Res Protoc. 2023 Dec 11;12:e50985. doi: 10.2196/50985 (PMC10750235; doi:10.2196/50985)
Supplement: Multimedia Appendix 1 [file resprot_v12i1e50985_app1.docx]

**World Bank list Low- income countries**

| Afghanistan | Korea, Dem. People's Rep | South Sudan |
| --- | --- | --- |
| Burkina Faso | Liberia | Sudan |
| Burundi | Madagascar | Syrian Arab Republic |
| Central African Republic | Malawi | ﻿﻿Togo |
| Chad | Mali | Uganda |
| Congo, Dem. Rep | Mozambique | Yemen, Rep. |
| Eritrea | Niger |  |
| Ethiopia | Rwanda |  |
| Gambia, The | Sierra Leone |  |
| Guinea-Bissau | Somalia |  |

**Lower- middle income countries**

| Angola | Jordan | Philippines |
| --- | --- | --- |
| Algeria | India | Samoa |
| Bangladesh | Iran, Islamic Rep | São Tomé and Principe |
| Benin | Kenya | Senegal |
| Bhutan | Kiribati | Solomon Islands |
| Bolivia | Kyrgyz Republic | Sri Lanka |
| Cabo Verde | Lao PDR | Tanzania |
| Cambodia | Lebanon | Tajikistan |
| Cameroon | Lesotho | Timor-Leste |
| Comoros | Mauritania | Tunisia |
| Congo, Rep. | Micronesia, Fed. Sts. | Ukraine |
| Côte d'Ivoire | Mongolia | Uzbekistan |
| Djibouti | Morocco | Vanuatu |
| Egypt, Arab Rep. | Myanmar | Vietnam |
| Eswatini | Nepal | Zambia |
| Ghana | Nicaragua | Zimbabwe |
| Guinea | Nigeria |  |
| Haiti | Pakistan |  |
| Honduras | Papua New Guinea |  |

**Upper-middle income countries**

| Albania | Fiji | North Macedonia |
| --- | --- | --- |
| Argentina | Gabon | Palau |
| Armenia | Georgia | Paraguay |
| Azerbaijan | Grenada | Peru |
| Belarus | Guatemala | Russian Federation |
| Belize | Indonesia | Serbia |
| Bosnia and Herzegovina | Iraq | South Africa |
| Botswana | Jamaica | St. Lucia |
| Brazil | Kazakhstan | St. Vincent and the Grenadines |
| Bulgaria | Kosovo | Suriname |
| China | Libya | Thailand |
| Colombia | Malaysia | Tonga |
| Costa Rica | Maldives | Türkiye |
| Cuba | Marshall Islands | Turkmenistan |
| Dominica | Mauritius | Tuvalu |
| Dominican Republic | Mexico | West Bank and Gaza |
| El Salvador | Moldova |  |
| Equatorial Guinea | Montenegro |  |
| Ecuador | Namibia |  |
