# Supplementary material for: Economic Burden of Mosquito-Borne Diseases in Low- and Middle-Income Countries: Protocol for a Systematic Review
Source: JMIR Res Protoc. 2023 Dec 11;12:e50985. doi: 10.2196/50985 (PMC10750235; doi:10.2196/50985)
Supplement: Multimedia Appendix 2 [file resprot_v12i1e50985_app2.docx]

**Search Strategy**

**Title:** Economic burden of mosquito borne diseases in low- and middle-income countries: A systematic review protocol

**Database:** MEDLINE

**Terms**: Cost and Mosquito borne disease and low- and middle-income countries

|  | **Mesh Terms** | **Text Words** |
| --- | --- | --- |
| Disease | |  |
| Chikungunya | Chikungunya Fevers[Mesh] |  |
| Dengue | Dengue[Mesh]  Severe Dengue[Mesh] |  |
| Lymphatic filariasis | Elephantiasis, Filarial[Mesh] |  |
| Rift Valley fever | Rift Valley fever[Mesh] |  |
| Yellow Fever | Yellow Fever[Mesh] |  |
| Zika | Zika Virus[Mesh]  Zika Virus Infection[Mesh] |  |
| Malaria | Malaria[Mesh] |  |
| Japanese encephalitis | Encephalitis, Japanese[Mesh] |  |
| West Nile fever | West Nile fever[Mesh] |  |
| Economic | |  |
| Cost | Economics[Mesh:NoExp]  Cost and Cost Anaysis[Mesh] | Costs and Cost Analysis[mh]  Economics, Nursing[mh] Economics, Medical[mh] Economics, Pharmaceutical[mh] Economics, Hospital"[mh] Economics, Dental[mh]  Fees and Charges[mh] Budgets[mh] budget*[tiab] economic*[tiab]  cost[tiab]  costs[tiab]  costly[tiab]  costing[tiab]  price[tiab]  prices[tiab]  pricing[tiab] pharmacoeconomic*[tiab] pharmaco-economic*[tiab] expenditure[tiab] expenditures[tiab]  expense[tiab]  expenses[tiab]  financial[tiab]  finance[tiab]  finances[tiab]  financed[tiab]  value for money[tiab]  monetary value*[tiab]  models, economic[mh] economic model*[tiab]  markov chains[mh] markov[tiab]  monte carlo method[mh]  monte carlo[tiab]  Decision Theory[mh]  decision tree*[tiab]  decision analy*[tiab]  decision model*[tiab]  Out of Pocket Expenditure [tiab]  OOP[tiab]  Catastrophic Expenditure[tiab] |
| LMIC | |  |
| Low- and middle-income country | Developing Countries[Mesh] | Africa[tiab]  Asia[tiab]  Caribbean[tiab]  West Indies[tiab]  South America[tiab]  Latin America[tiab]  Central America[tiab]  Afghanistan[tiab]  Angola[tiab]  Armenia[tiab]  Armenian[tiab]  Bangladesh[tiab]  Benin[tiab]  Bhutan[tiab]  Bolivia[tiab]  Burkina Faso[tiab]  Burkina Fasso[tiab]  Burundi[tiab]  Cambodia[tiab]  Central African Republic[tiab] Chad[tiab]  Comoros[tiab]  Congo[tiab]  Cote d'Ivoire[tiab]  Ivory Coast[tiab]  Djibouti[tiab]  Egypt[tiab]  El Salvador[tiab]  Eritrea[tiab]  Ethiopia[tiab]  Gambia[tiab]  Gaza[tiab]  Georgia[tiab]  Ghana[tiab]  Guatemala[tiab]  Guinea[tiab]  Guam[tiab]  Haiti[tiab]  Honduras[tiab]  India[tiab]  Indonesia[tiab]  Kenya or Kiribati[tiab]  Korea[tiab]  Kosovo[tiab]  Kyrgyzstan[tiab]  Lao PDR[tiab]  Lesotho[tiab]  Liberia[tiab]  Madagascar[tiab]  Malawi[tiab]  Mali[tiab]  Mauritania[tiab]  Moldova[tiab]  Mongolia[tiab]  Morocco[tiab]  Mozambique[tiab]  Myanmar[tiab]  Myanma[tiab]  Nepal[tiab]  Nicaragua[tiab]  Niger[tiab]  Nigeria[tiab]  Pakistan[tiab]  Paraguay[tiab]  Philippines[tiab]  Philipines[tiab]  Phillipines[tiab]  Phillippines[tiab]  Rwanda[tiab]  Ruanda[tiab]  Sao Tome[tiab]  Senegal[tiab]  Sri Lanka[tiab]  Solomon Islands[tiab]  Somalia[tiab]  Sudan[tiab]  Swaziland[tiab]  Tajikistan[tiab]  Tanzania[tiab]  Timor-Leste[tiab]  Tokelau[tiab]  Togo[tiab]  Tuvalu[tiab]  Uganda[tiab]  Ukraine[tiab]  Uzbekistan[tiab]  Vanuatu[tiab]  Vietnam[tiab]  Viet Nam[tiab]  West Bank[tiab]  Yemen[tiab]  Zambia[tiab]  Zimbabwe[tiab]  Less Developed Countries[tiab]  Under Developed Countries[tiab]  Low Income Country[tiab]  Middle Income Countries[tiab]  LMICs[tiab]  Third-World Countries[tiab] |
