# Supplementary material for: Economic Burden of Mosquito-Borne Diseases in Low- and Middle-Income Countries: Protocol for a Systematic Review
Source: JMIR Res Protoc. 2023 Dec 11;12:e50985. doi: 10.2196/50985 (PMC10750235; doi:10.2196/50985)
Supplement: Multimedia Appendix 3 [file resprot_v12i1e50985_app3.docx]

**Data Extraction Form**

| S.No | Category | Sub Category | Notes |
| --- | --- | --- | --- |
|  | Source | Study ID, Year |  |
|  | Study objective(s)/ Research question | |  |
|  | Study methods | Study design  Type of Economic Evaluation  Study setting  Study period  Sample size |  |
|  | Participant Characteristics | Total number  Age  Country  Region/State/District |  |
|  | Disease |  |  |
|  | Costing characteristics | Costing year, Inflation adjustment, Currency unit, Currency conversion rate to USD |  |
|  | Cost components | Direct medical cost, Direct non-medical cost and Indirect cost |  |
|  | Direct medical cost |  |  |
|  | Direct non-medical cost |  |  |
|  | Indirect cost |  |  |
|  | Cost methodology | Bottom-up costing, Top-down costing, micro-costing, gross-costing |  |
|  | Miscellaneous | Key conclusions of study authors  Limitations of the study  Miscellaneous comments from study authors  Funding source  Conflict of interest,  References to other relevant studies |  |
|  | Results |  |  |
|  | Conclusion |  |  |
